# Novel behavioral indicator of explicit awareness reveals temporal course of frontoparietal neural network facilitation during motor learning

**DOI:** 10.1371/journal.pone.0175176

**Published:** 2017-04-14

**Authors:** Regan R. Lawson, Jordan O. Gayle, Lewis A. Wheaton

**Affiliations:** 1 School of Biological Sciences, Georgia Institute of Technology, Atlanta, Georgia, United States of America; 2 School of Psychology, Morehouse College, Atlanta, Georgia, United States of America; Universita degli Studi di Roma La Sapienza, ITALY

## Abstract

Deficits in sequential motor learning have been observed in many patient populations. Having an understanding of the individual neural progression associated with sequential learning in healthy individuals may provide valuable insights for effective interventions with these patients. Due to individual variability in motor skill acquisition, the temporal course of such learning will be vary, suggesting a need for a more individualized approach. Knowing when a subject becomes aware of movement patterns may provide a marker with which to identify each individual’s learning time course. To avoid interfering with the incidental nature of discovery during learning, such an indicator requires an indirect, behaviorally-based approach. In Part I, our study aimed to identify a reliable behavioral indicator predictive of the presence of incidental explicit awareness in a sequential motor learning task. Part II, utilized the predictive indicator and EEG to provide neural validation of perceptual processing changes temporally correlated with the indicator. Results of Part I provide a reliable predictive indicator for the timing of explicit awareness development. Results from Part II demonstrates strong classification reliability, as well as a significant neural correlation with behavior for subjects developing awareness (EXP), not observed with subjects without awareness (NOEXP). Additionally, a temporal correlation of peak activation between neural regions was noted over frontoparietal regions, suggesting that the incidental discovery of motor patterns may involve a facilitative network during awareness development. The proposed indicator provides a tool in which to further examine potential impacts of awareness associated with incidental, or exploratory, motor learning, while the individual nature of the indicator provides a tool for monitoring progress in rehabilitative, exploratory motor learning paradigms.

## Introduction

Sequential motor learning is pivotal for successful execution of many everyday activities. Sequential motor learning deficits have been demonstrated with aging, as well as in stroke patients and amputees [1–3]. Having an understanding of the neural changes associated during sequential motor learning in healthy individuals may provide insights into potential interventions for such populations.

Skilled motor learning is proposed to involve both an implicit and explicit system. The implicit system utilizes exposure to repetitive movements for movement optimization, while the explicit system is involved in identifying and planning the overarching task goal with conscious awareness [4]. The serial reaction time task (SRTT) is a paradigm often utilized to examine both behavioral and neural changes associated with sequential motor learning. In this paradigm, subjects quickly match response locations to presented visual stimuli, while experiencing an embedded sequence [5]. Over the experiment, subject response times are noted to decrease due to the embedded sequence. Due to the typical lack of awareness, improved latencies are attributed to the implicit system optimization, with the explicit system being restricted to the task goal of pressing the appropriate button [6]. While most subjects remain unaware of the sequence, some subjects develop explicit awareness of the sequence, but are often excluded from further analysis. Recent work, however, has demonstrated beneficial impacts associated with the incidental development of awareness, including enhanced perceptual sensitivity and motivational vigor [7, 8].

While these incidental awareness studies, necessarily, assessed if awareness was present, they did not attempt to predict *when*, during the experiment, such awareness occurred. It is known that variations in skill learning occur, often attributed to a subject’s initial state [9]. This variation could result in time course differences in awareness development, potentially hiding valuable information when reporting group averages. One method to address this concern is to examine learning-dependent changes normalized to each individual’s point of discovery. Such an approach necessitates the use of an indicator which detects, not only if awareness is present, but also when it occurred. This, however, is not a trivial task, as the indicator must be one which can detect awareness onset while avoiding interference with the incidental nature of discovery. Therefore, the indicator must be determined indirectly through behavioral measures such as changes in response latencies.

Awareness is shown to promote a behavioral strategy shift allowing for anticipatory movements, resulting in faster overall response latencies [10]. This strategy change may be reflected neurally as a shift from an externally-guided network, relying on visual stimuli for motor execution, to a more internally-guided network due to knowledge of upcoming response locations. Additionally, as explicit awareness development has been shown to result in improved perceptual sensitivity [7], it is expected that awareness will also involve alterations in the perceptual processing of visual stimuli. Therefore, a reliable behavioral indicator of awareness would be one which demonstrates a significant temporal correlation with these neural changes.

To this end, the current study employed event-related EEG analyses to examine perceptual activation changes occurring during sequential learning to provide neural assessment of the proposed behavioral predictor. Previous studies examining neural regions involved in implicit sequential learning have demonstrated a frontoparietal network including sensorimotor, parietal and premotor cortices [11], with prefrontal and supplementary motor areas being additionally recruited for explicit learning and awareness [12]. Therefore, it is expected that subjects developing awareness will demonstrate a shift in neural activation reflective of explicit learning.

The current study tests two hypotheses: (1) significant changes in latency, relative to individual baseline performance, will be predictive of awareness; (2) neural changes seen for explicitly aware subjects will correlate with behavioral changes identified by the indicator that are not present for subjects failing to develop awareness.

## Materials and methods

### Experiment 1: Model development of predictive indicator

#### Subjects

Thirty right-handed (age 18–31 years; 13 females), neurologically healthy adults were recruited to participate in the study. All subjects provided written, informed consent, and the Georgia Institute of Technology Institutional Review Board approved all methods. Subjects completed an Edinburgh Handedness Inventory [13] to assess the level of handedness along with a short questionnaire regarding any previous musical training they had received. Only subjects with a handedness score greater than 0.6 (indicating right hand dominance), and less than 3 years formal musical training, were included from the study.

#### Experimental paradigm

Subjects were randomly placed in one of three groups, each experiencing a different number of elements of a sequence: (1) 7-element sequence (7-KEY), (2) 10-element sequence (10-KEY), and (3) 13-element sequence (13-KEY). Previous studies examining incidental awareness have utilized sequences of 6–8 elements in length, while studies examining motor learning without awareness utilized sequences greater than 10 elements [14–17]. Therefore, the authors utilized the selected sequence lengths of 7-, 10-, and 13-elements to ensure some subjects will develop awareness, while other subjects remain unaware of the sequence during the study. Due to the focus on the development of incidental explicit awareness, a within-subject design between sequence lengths was not possible due to the potential confound of expecting a repetitive pattern after the first sequence exposure. The sequences were generated in a pseudorandom fashion by a MATLAB code (Natick, MA) to ensure that no consecutive sequence elements were repeated and 3 consecutive fingers would not be used in the sequence. A modified serial reaction time task (SRTT) was used, with no random sequence at the end of the paradigm to allow for sequence recall assessment. Visual stimuli consisted of four white rectangles equally spaced in a horizontal manner on the screen. To start the experiment, one of the four rectangles would change from white to red. Subjects were asked to match the relative location of the red rectangle to the location on the 4-button response pad as quickly and accurately as possible. Using their right hand to respond, this corresponded to subjects using the index finger for the far most left rectangle and the far most right rectangle matching to the little finger. Stimulus presentation and timing was managed utilizing STIM2 software from Compumedics Neuroscan^™^ (Charlotte, NC). Subjects had 750 msec in which to record a response, at which time the next visual stimuli would immediately appear providing an inter-stimulus interval (ISI) of 0 msec. Subjects in the 7- and 13-element conditions were exposed to 182 stimuli per block, with the 10-element condition receiving 180 stimuli per block. This provided each condition a complete number of sequences within each block. Subjects experienced a total of 5 blocks, with a 30 second rest between blocks. 4 seconds before the end of the rest period, subjects received a 3-sec “Get Ready” visual cue and a 1-sec fixation cross, followed by the next block of visual stimuli. Online data collection included latency and accuracy of each response. Upon completion of the paradigm, subjects were asked if they noticed a pattern within the presentation. If they answered yes, subjects were asked to replicate the pattern noticed. Replication responses were recorded to assess for accuracy. Subjects able to repeat the sequence with 100% accuracy were identified as explicitly aware, EXP. As some subjects may learn the sequence at a different starting point than what was presented, accuracy was not limited to the presented starting point. For example, a subject experiencing the 7-element sequence 3-4-2-1-3-2-4, would be classified as EXP if their recall was 3-4-2-1-3-2-4 or if it was 2-1-3-2-4-3-4. Subjects unable to repeat the sequence with 100% accuracy were categorized as not explicitly aware, NOEXP for the purpose of model development. Offline analysis examined latency and accuracy changes for all subjects throughout the experiment. Individual subject data was averaged for each sequence repetition, consisting of 7-, 10-, or 13-key presses.

#### Statistical analyses of behavioral data

Individual subject data was analyzed utilizing a custom MATLAB (Natick, MA) program. Subject data was first separated into sequence repetitions. To ensure an equal number of sequences for analysis between conditions, analysis was only conducted for the first 70 sequence repetitions, which represented the maximal number of repetitions experienced by the 13-KEY condition. Removal of anticipatory or late key presses (an average of 15% of responses) allowed for analysis of only recorded responses for both accuracy and latency changes. Individual average accuracy and latency of recorded responses for each trial was calculated and utilized for group comparisons. Individual subject baseline performance was calculated as the mean of the first 45 key presses, with the standard deviation being used as a variable in examining predictive thresholds. A repeated-measures analysis of variance (ANOVA) was conducted, utilizing R stats [18], for both accuracy and latency, with SEQ (70 levels) as within-subject, repeated measures variables and CONDITION (3 levels) and AWARENESS (2 levels) as between-subject variables, with significance levels reported at p < 0.05, and Holm correction for multiple comparisons. When sphericity was violated, Greenhouse-Geisser corrected p-values are reported.

#### Development of a model for individualized threshold predictive of explicit awareness

Individual performance at baseline, defined as the first trial of 45 keys, was utilized to calculate the individualized threshold. Behaviorally, a subject’s initial state has been attributed to variations in skill learning suggesting a need to individualize the indicator [9]. Therefore, neurobehavioral changes relative to individual baseline performance was hypothesized to provide a stronger basis for evaluating correlates to incidental explicit awareness. Behaviorally, it was expected that subjects demonstrating latencies significantly different than baseline behavior would be indicative of the presence of awareness of the sequence. For this reason, the behavioral threshold was calculated based on a subject’s baseline mean and standard deviation, allowing for a z-score threshold analysis. The formula utilized to test the reliability of an individualized threshold in classifying subjects as having explicit awareness is shown in Eq 1

$$\begin{array}{c} \text{Threshold}={Mean}_{\text{baseline}}-(z\text{-}score)*({SD}_{\text{baseline}}) \end{array}$$ (1)

A customized MATLAB program was then utilized to classify subjects as having explicit awareness (EXP) or no explicit awareness (NOEXP) based on their mean performance for each sequence repetition (ie. 7 keys for the 7-element, 10 keys for the 10-element and 13 keys for the 13-element condition) over the course of the experiment compared to the calculated threshold. To improve reliability of using the subject mean value for each trial, the upper confidence interval was determined as the sequence behavior in which to compare against the calculated threshold. If a subject’s upper confidence interval for a sequence repetition dropped below the calculated threshold at any point during the experiment, the classifier would identify the subject as EXP. If the upper confidence interval did not drop below the calculated threshold at any point during the experiment, the classifier identified the subject as NOEXP. The classification results were then compared to the actual experimental results for EXP and NOEXP subjects to determine the sensitivity, or true positive rate (TPR), and specificity, or true negative rate (TNR), of the classifier for that z-score value. Calculations for sensitivity and specificity are shown in Eqs 2 and 3 respectively. Z-score values ranging from 0.5 to 2.5, in increments of 0.01, were examined to identify the most reliable z-score in predicting a subject as having explicit awareness. Sensitivity/specificity analysis was then also conducted utilizing an additional constraint of performing for two consecutive sequence repetitions below threshold for each z-score value to examine the predictive efficacy of a consistency requirement:

True positive (TP) = accurate EXP classification
False negative (FN) = inaccurate NOEXP classification
True negative (TN) = accurate NOEXP classification
False positive (FP) = inaccurate EXP classification

$$\begin{array}{c} \text{Sensitivity}(\text{TPR})=\text{TP}/(\text{TP}+\text{FN}) \end{array}$$ (2)

$$\begin{array}{c} \text{Specificity}(\text{TNR})=\text{TN}/(\text{TN}+\text{FP}) \end{array}$$ (3)

### Experiment 2: Neurobehavioral assessment of the individualized threshold model

#### Subjects

An additional twenty-one right-handed (age 18–32 years; 9 females), neurologically healthy adults were recruited to participate in Experiment 2. All subjects provided written, informed consent, and the Georgia Institute of Technology Institutional Review Board approved all methods. As in Experiment 1, subjects completed an Edinburgh Handedness Inventory [13] to assess the level of handedness along with a short questionnaire regarding any previous musical training they had received.

#### SRTT behavioral task

Based on the results of Experiment 1, Experiment 2 focused solely on the 7-element sequence. Subjects experienced the same sequence as those in Experiment 1, but a customized MATLAB program was written utilizing PsychToolBox [19] for presentation to allow for the recording of anticipatory responses and stimulus markings necessary for EEG epoching. A small black box appeared in the upper and lower left corners of the screen to allow for a photodiode impulse to be detected by StimTracker^™^ (Cedrus Corporation, San Pedro, CA). A pulse was transmitted both at the onset of each stimulus and at the beginning of each block. This adjustment provided the opportunity to record all responses from EXP subjects and had the potential to result in mean sequence latencies that were negative relative to the stimulus onset. Behavior analysis focused on latency and accuracy of responses. Based on observations from Experiment 1, a 35-key random sequence was presented in the first block and utilized to calculate each subject’s individualized threshold. Based on the sensitivity/specificity examination in Experiment 1, threshold calculations were made based on a z-score of −1.85. Following the baseline block, subjects experienced 26 blocks consisting of (5) repetitions of the 7-element sequence. This provided a total of 130 sequence repetitions to match the number of sequence repetitions presented in Experiment 1. The stimulus presentation time of 750 ms and ISI of 0 ms was also maintained for consistency. Subjects experienced a 10-second rest period between blocks, with a 30-second rest period after blocks 10 and 20. Upon completion of the experiment, subjects were asked if they recognized a pattern during the experiment and then asked to replicate the pattern. As in Experiment 1, subjects able to replicate the sequence with 100% accuracy were identified as EXP, while those unable to replicate the sequence with 100% accuracy were identified as NOEXP.

#### EEG recording and pre-processing

EEG analysis utilized a 58-channel EEG cap (Electrocap, Eaton, OH) that recorded scalp potential activity (1000 Hz sampling rate, filtered at DC-100 Hz) via the SynAmps II^™^ data acquisition system (Compumedics Neuroscan, Charlotte, NC). Data were referenced to the ear electrode and impedances kept below 5 k. The raw, continuous EEG data was then imported into MATLAB’s EEGLAB [20] for pre-processing and analysis. Data was first high-pass filtered at 0.5 Hz, bad channels were identified, rejected and interpolated. Data was then epoched from 100 ms pre- stimulus onset to 500 ms post-stimulus for all 945 stimuli presentations with baseline correction from 100 to 0 msec, to provide epochs for subsequent event-related potential (ERP) analysis. An independent component analysis (ICA) was then conducted, utilizing EEGLAB’s *runica* algorithm, to assist in removal of blink and other stereotypical movement artifact components. Selection of components for removal was based on visual inspection of scalp map localization, unusual spectral frequency patterns and irregular ERP-image activity. An average of 7 of 58 components per participant was selected for subtraction. After removal of artifact components, data was segmented into 27 separate datasets, one for each block. A separate dipole fitting analysis was then conducted, utilizing EEGLAB’s DIPFIT plugin [21], for every block for each subject. Dipole localization was determined utilizing the Talairach Client application [22]. After pre-processing, all datasets were loaded into a STUDY structure for group analysis.

#### Behavioral validation of threshold model

Post-experimental analysis converted raw latency values to individualized z-scores both for awareness classification and statistical comparison between subjects. A customized MATLAB program was utilized to calculate the sequence repetition at which a participant had achieved two consecutive repetitions at a performance level below their individualized threshold z-score value of −1.85 and were classified as EXP. Participants failing to drop below threshold were classified as NOEXP. A sensitivity/specificity analysis was again conducted comparing actual vs. predicted classification of awareness. In addition, for EXP subjects, the block at which behavior dropped below threshold was identified as the block of interest for EEG correlation analysis, while the block of lowest z-score performance was utilized as the block of interest for the NOEXP subjects. Subjects who demonstrated full recall without dropping below threshold were identified as false negatives (FN) and excluded from further statistical analysis due to the low number of subjects in this category.

#### EEG statistical and correlation analysis

Subject datasets were loaded into EEGLAB Study design with identification of the between subject group classification of awareness (EXP vs. NOEXP) and within subject condition of time (Blocks 1–27). Group analyses were conducted by first grouping the data into 9 sections of 3 blocks each. ERP group measures were precomputed utilizing the baseline of −100 to 0 ms. A repeated measures ANOVA was conducted, utilizing permutation statistics with false discovery rate (FDR) correction for multiple comparisons (alpha = 0.05), with BLOCK (27 levels) as within-subject, repeated measures variables and AWARENESS (2 levels) as between subjects variables. AWARENESS categories consisted of EXP (100% recall and correctly classified as EXP by the previously defined threshold) and NOEXP (<100% recall and correctly classified as NOEXP by the threshold). Specific electrode locations were determined by first examining the ERP scalp map distribution of time periods based on prior work for the ERP components of interest [23]. These post-stimulus time periods included: (1) early N1: 100 to 150 ms, (2) late N1: 150 to 200 ms, (3) P2: 150 to 250 ms, and (4) P3: 250 to 400 ms. Upon examination of the scalp map distributions, specific electrode locations were selected based on the parieto-frontal network noted to be involved in visuospatial motor tasks through multiple studies [24–28]. These electrode combinations included: (1) early N1 frontal electrodes (F1, FZ, F2), (2) late N1 parietal electrodes (P1, P3, P2, P4), (3) P2 left frontocentral electrodes, due to the right-handed nature of the task (C1A, CZA, C1, CZ), and (4) P3 centroparietal electrodes (C1, CZ, C2, C1P, PZA, C2P). Time ranges utilized in statistical analysis were narrowed post hoc based on the range limits demonstrated by subjects for each component of interest. This resulted in the following time windows: (1) early N1: 100–140 ms, (2) late N1: 140–170 ms, (3) P2: 180–210 ms, and (4) P3: 275–375 ms. Component amplitude values for each block were determined utilizing the signed area amplitude method [23]. The method of signed area amplitude was utilized to address the possible confound of amplitude magnitude differences on subsequent components of the ERP. Corrections for multiple comparisons in the group analysis of ERP components were conducted utilizing EEGLAB’s false discovery rate and permutation sampling method. Adjusted p values are reported in results.

As the behavioral indicator provided a prediction of awareness for a specific block, correlations between individual behavioral performance and neural amplitude for each ERP component were conducted with data from all 27 blocks. The behavioral performance variable, for EXP subjects, was the block at which behavior was indicative of awareness by the predictive indicator. As NOEXP subjects had no behavior indicative of awareness, the block at which the lowest z score was observed was utilized as the behavioral performance variable. The neural behavior variable was the block at which the largest amplitude occurred. As the P3 amplitude has been noted to be influenced by pacing attributes [29], it was expected that an initial peak would occur during the first 2 blocks relative to adaptation of the stimulus-response pacing, Therefore, analysis of changes in peak amplitude was limited to Blocks 3–27. A Spearman correlation analysis conducted was then conducted for peak amplitude relative to behavioral performance for each component. An additional correlation for P300 amplitude vs. response latency over the course of the experiment was conducted to explore the correlation of behavioral latency on the P300 peak amplitude for both EXP and NOEXP groups utilizing a linear mixed model. Fixed effects were behavioral latency and awareness, with an interaction term, and random effects of intercepts for subjects. Correlation analysis was conducted utilizing R statistics packages *rcorr* and *lmer* [30]. Multiple comparisons for correlations were addressed utilizing Holm corrections and adjusted p values reported.

## Results

### Results: Experiment 1

#### Subject demographics

There was no significant difference in age, handedness, or years of previous musical experience between sequence conditions or awareness classification (see Table 1).

**Table 1:** Subject demographics.

Condition	Age (yrs)	Handedness	Formal Musical Training (yrs)
7 − *Element*	22.0 ± 1.01	0.88 ± 0.04	1.5 ± 0.40
10 − *Element*	22.3 ± 1.45	0.88 ± 0.04	1.2 ± 0.42
13 − *Element*	22.0 ± 1.41	0.90 ± 0.03	1.7 ± 0.56
*Experiment*2: *EXP*	19.8 ± 1.23	0.92 ± 0.10	1.3 ± 1.33
*Experiment*2: *NOEXP*	21.4 ± 3.95	0.97 ± 1.33	1.2 ± 1.33

#### Effect of sequence length: Accuracy

The mean accuracy for recorded responses was 97.93% ± 6.61%. One subject from the 13KEY condition experienced problems with the response pad for multiple blocks throughout the experiment. Therefore, the 13KEY condition data analysis is restricted to 9 subjects. Two additional subjects, one from the 7KEY and one from the 10KEY, each had one sequence repetition, repetition 13 and repetition 15, respectively, which did not record multiple responses. The mean latency from the previous repetition was, therefore, used to represent the missing data for these subjects. A repeated measures ANOVA of accuracy for SEQxCONDITION, showed no significant difference for sequence repetition (F(69, 1799) = 1.048, p = 0.373) or between conditions of sequence length (F(2, 1799) = 0.963, p = 0.382) as shown in Table 2.

**Table 2:** Mean baseline latency & accuracy by sequence lengths.

Measure	7-Element	10-Element	13-Element
*BaselineLatency*(*msec*)	458.69 ± 51.76	451.80 ± 34.61	438.69 ± 28.09
*Accuracy*	98.3% ± 0.27%	98.08% ± 0.29%	97.94% ± 0.54%

#### Effect of sequence length: Accuracy

The mean latency for accurate recorded responses, at baseline, for all subjects, was 453.41 ± 29.42 msec, with an average final latency of 346.46 ± 68.35 msec. The baseline latency performance for all subjects showed no significant difference between conditions. In line with previous SRTT studies [5, 14, 31], all subjects demonstrated a decrease in z-score, relative to baseline latency, over the 70 sequence repetitions experienced. Repeated measures ANOVA for CONDITION, with Greenhouse-Geisser correction, indicated a main effect of sequence repetition for change of z-score (F(69, 1799) = 3.908, p<0.001), a main effect of sequence length (F(2, 1799) = 53.368, p<0.001), with no interaction effect of sequence length condition for z-score change over the 70 sequences for conditions, (F(138, 1799) = 0.547, p = 1). Simple main effects analysis showed significant effects (p<0.05) appearing between 7-element and 10-element conditions by sequence 69, between 7-element and 13-element by sequence 9, and between 10-element and 13-element conditions by sequence 21.

#### Effect of explicit awareness

Eight subjects were able to correctly replicate the sequence at the end of the experiment and were identified as having explicit awareness, EXP (7 subjects from the 7-KEY condition; 1 subject from the 13-KEY condition). Twenty-one subjects were unable to correctly replicate the entire sequence and were identified as having no explicit awareness, NOEXP. Explicit recall by sequence length summary is shown in Table 3.

**Table 3:** Percent recall by sequence length.

Condition	Number of EXP Subjects	Percentage of Recall for NOEXP Subjects
7 − *Element*	7/10	42.86% ± 11.66%
10 − *Element*	0/10	17.00% ± 21.93%
13 − *Element*	1/10	7.69% ± 14.50%

#### Effect of explicit awareness: Accuracy

Accuracy of recorded responses was 98.10% ± 0.28% for the EXP subjects and 98.24% ± 0.33% for the NOEXP subjects. A repeated measures ANOVA of accuracy for SEQxAWARENESS showed no significant difference of sequence repetition (F(69, 1869) = 1.045, p = 0.379) or between conditions of awareness (F(1, 1869) = 0.541, p = 0.460).

#### Effect of explicit awareness: Latency

**Fig 1:**
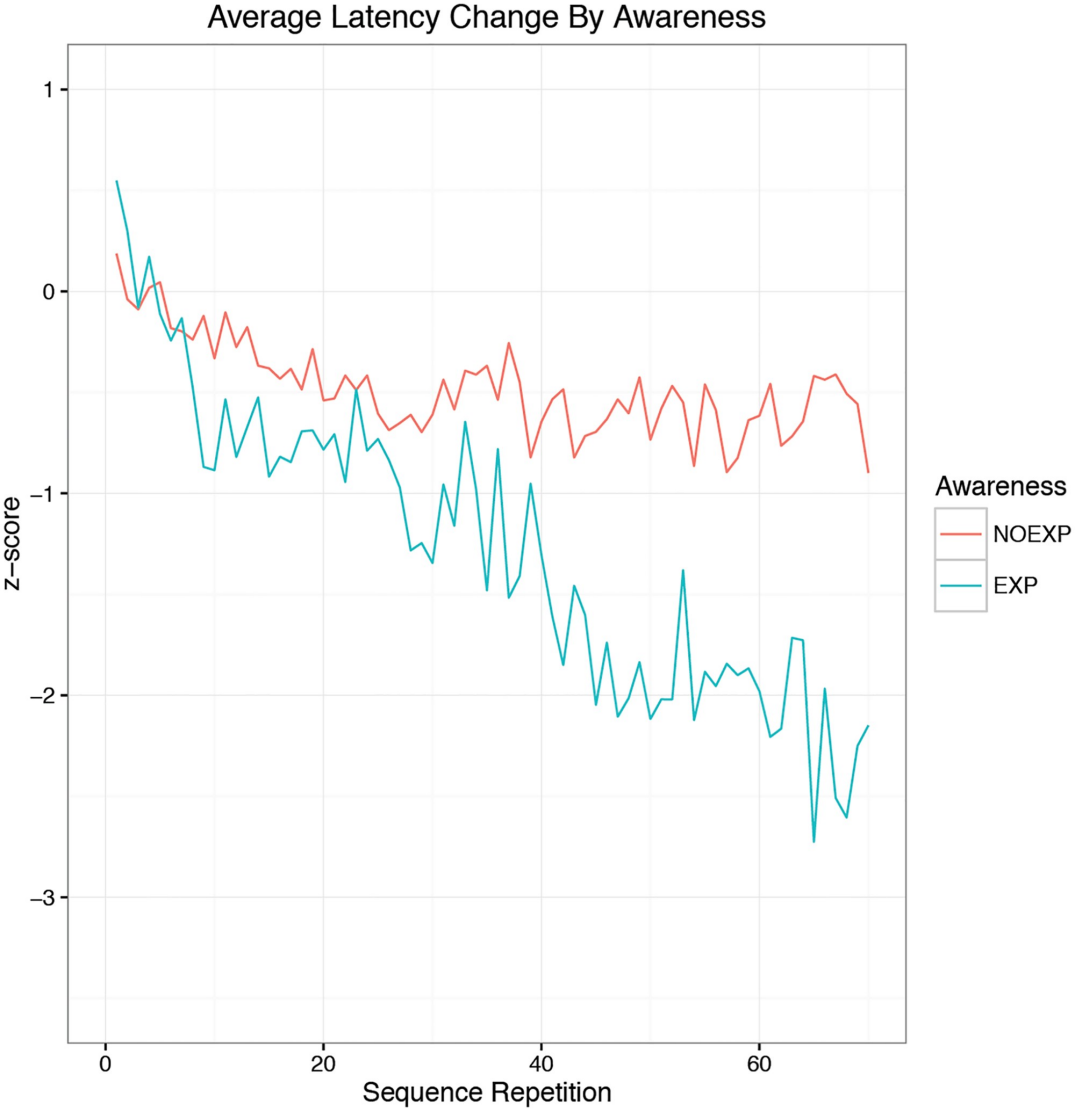
Latency change by awareness. Latency change over time for subjects with (EXP) or without (NOEXP) sequence awareness.

There was no significant difference between EXP and NOEXP conditions for baseline performance (F(27, 1) = 3.863, p = 0.0597, EXP = 471.12 ± 34.26 ms, NOEXP = 442.10 ± 38.76 msec). Fig 1 shows the z-score change in latency by awareness, demonstrating the significant difference in response times for subjects expressing EXP recall. Repeated measures ANOVA for AWARENESS, with Greenhouse-Geisser correction, demonstrated a significant main effect of sequence repetition for z score (F(68, 1869) = 4.897, p<0.001), a significant main effect of awareness (F(1, 1869) = 389.749, p<0.001) and a significant interaction effect (F(68, 1869) = 3.245, p<0.001). Simple main effects analysis showed significant effects (p<0.05) appearing between EXP and NOEXP subjects by sequence 37, continuing for the remainder of the experiment.

#### Reliability of the individualized threshold in predicting the presence of explicit awareness

**Fig 2:**
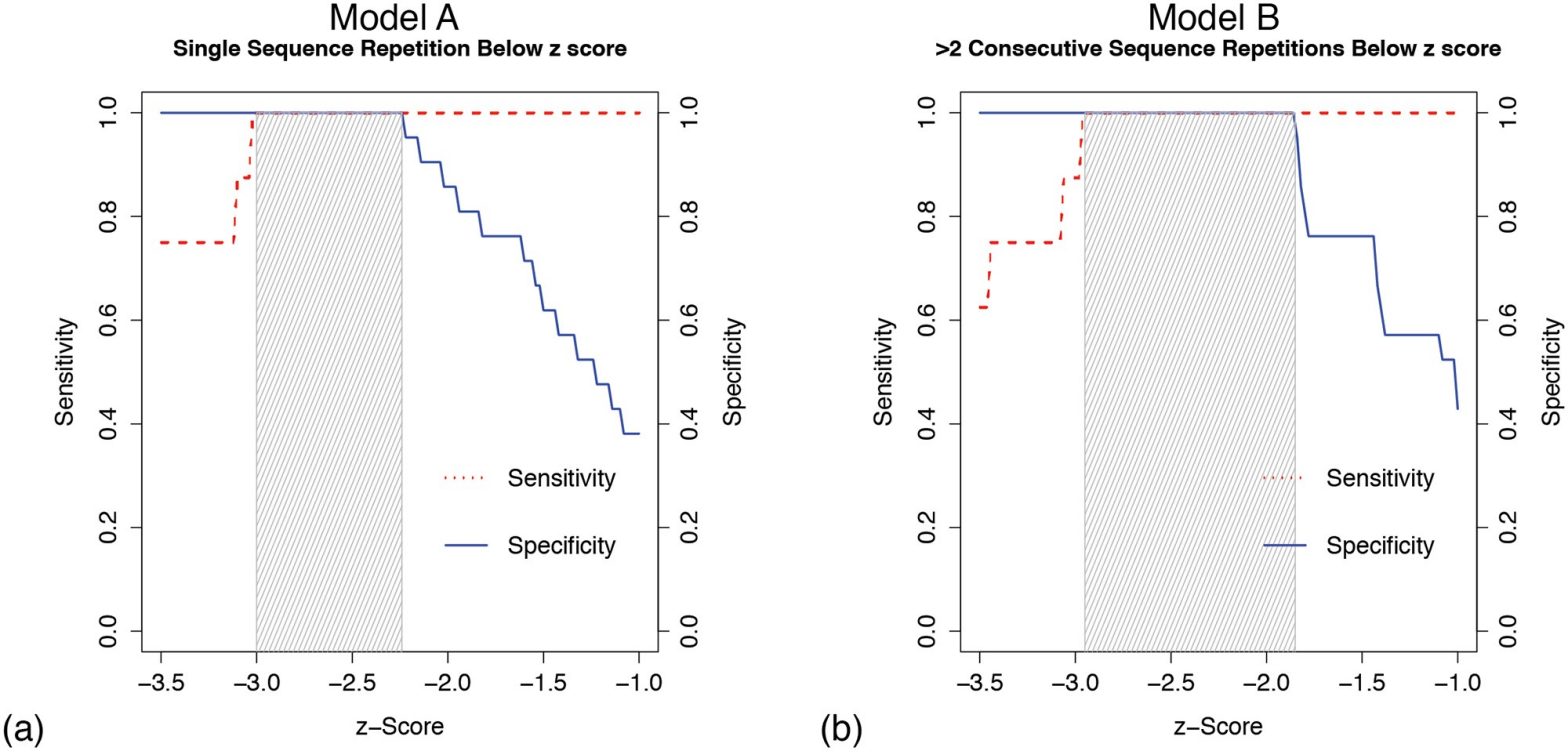
Sensitivity/Specificity plot for threshold model. (a) Classification of EXP based on the first occurrence of a sequence repetition with 95% confidence level performance below threshold. (b) Classification of EXP based on two consecutive occurrences of a sequence repetition with 95% confidence level performance below threshold.

The results of the sensitivity/specificity analysis for the threshold model are in shown in Fig 2. Fig 2a shows results when utilizing the first occurrence of behavior below the individualized threshold, while Fig 2b shows the requirement of two consecutive sequence occurrences below threshold. There is a distinct improvement in the rigor of accurately classifying a subject as EXP when the threshold model requires two consecutive sequence occurrences below threshold. Utilizing this model, it was noted that a minimum z score of −1.85 was required to provide a 100% sensitivity and specificity in accurately classifying a subject as EXP or NOEXP.

### Results: Experiment 2

#### Behavioral results: Reliability of threshold model

Eleven (11) subjects successfully recalled the sequence, with nine of them correctly identified as explicit (EXP) by the threshold model and two subjects incorrectly classified as non-explicit, giving a false negative classification (FN). The remaining ten (10) subjects were unable to accurately recall the sequence and were correctly identified as non-explicit (NOEXP). This results in a sensitivity of 81.8% and specificity of 100% for the individualized threshold model.

#### Behavioral results: Accuracy

Accuracy of the baseline block was 95.87% ± 3.23% for the EXP group and 96.86% ± 3.42% for the NOEXP group, while the overall accuracy for the 26 sequence blocks was 96.63% ± 2.34% for the EXP group and 96.31% ± 2.29% for the NOEXP group. A repeated measures ANOVA of accuracy for TIMExAWARENESS showed no main effect of time (F(1, 15) = 0.233, p = 0.637) or between conditions of awareness(F(1, 15) = 0.178, p = 0.679).

#### Behavioral results: Latency

**Fig 3:**
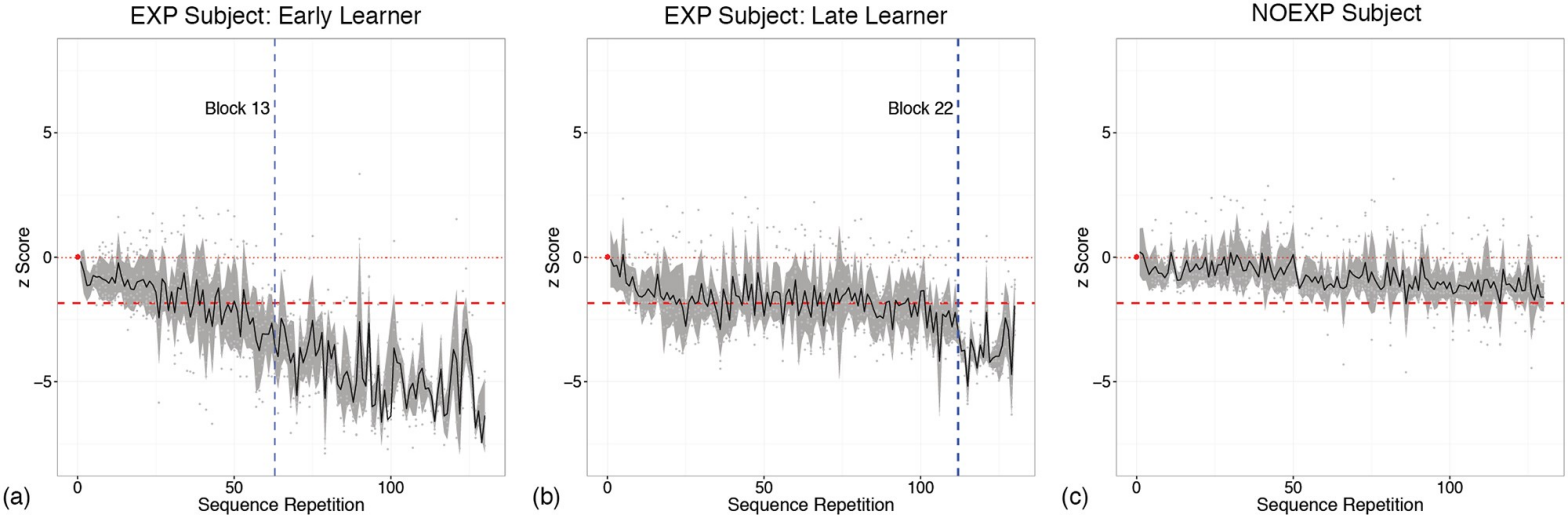
Representative individual performance graphs for the aware (EXP) and unaware (NOEXP) groups. The thick, dashed red line indicates each subject’s individualized baseline-referenced threshold calculated at a z-score of −1.85. The vertical blue line (3a and 3b) indicate the block at which EXP subjects demonstrated two consecutive sequence repetitions with performance below threshold. Although Subject 16 shown in 3c did not demonstrate behavior below threshold, there was a consistent decrease in latency over the experiment suggestive of implicit sequential learning.

**Fig 4:**
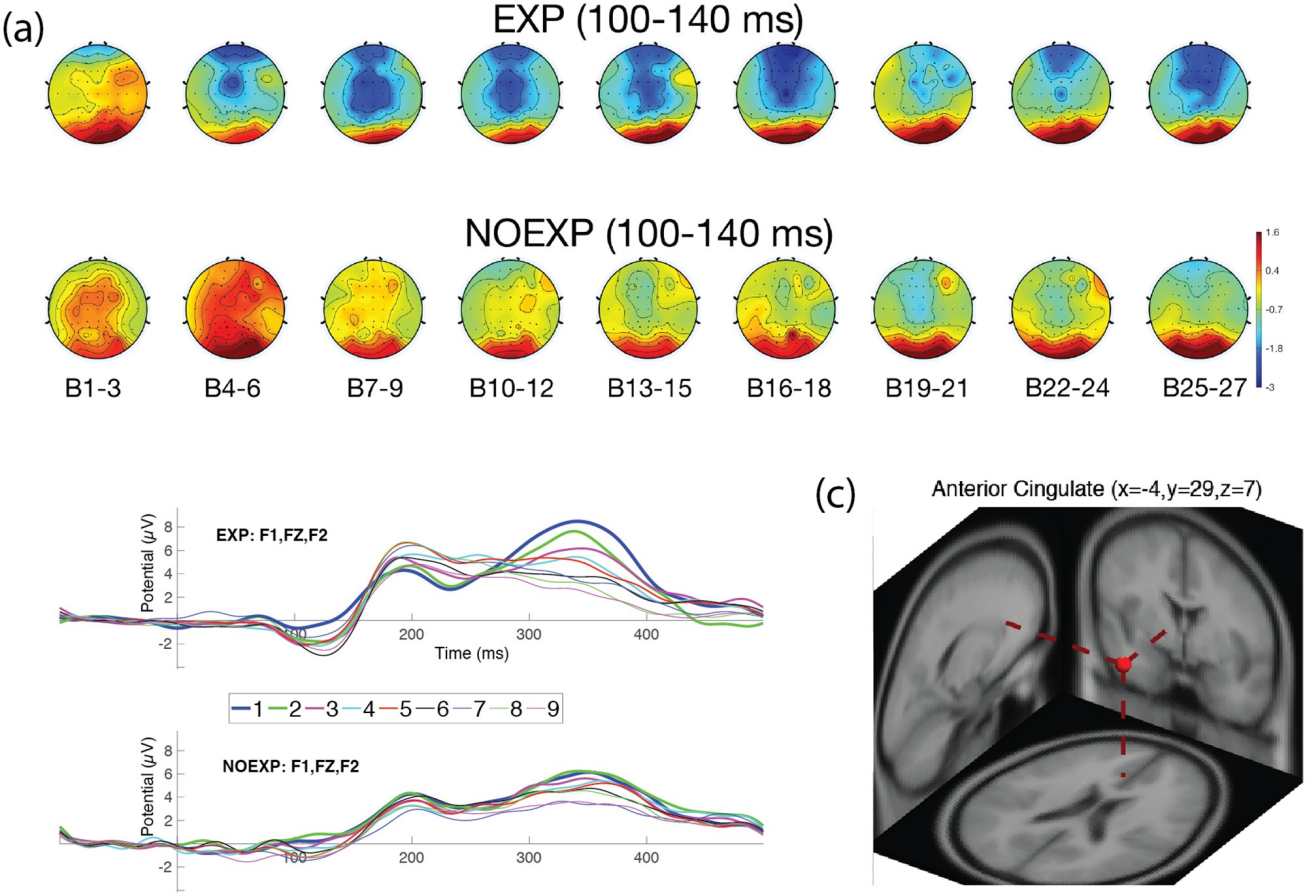
Neural activity for early N1 over frontal region. (a) Head maps for 9 periods of time during early N1 (100–140 msec). (b) ERP graph over frontal electrodes (F1, FZ, F2). (c) Dipole localization for early N1 component over anterior cingulate region.

Baseline latency showed a significant difference for awareness (F(666, 1) = 7.938, p = 0.005), with EXP subjects having a faster baseline mean latency (EXP = 357.93 ± 85.35 msec, NOEXP = 378.93 ± 100.52 msec). A repeated measures ANOVA of z score performance for TIMExAWARENESS demonstrated a main effect of time (F(25, 415) = 11.0280, p<0.001) and awareness (F(1, 415) = 311.6309, p<0.001) with an interaction effect of awareness (F(25, 415) = 3.9422, p<0.001). Final z scores showed a decrease of −4.055 ± 2.228 for the EXP group and −1.132 ± 0.619 for the NOEXP group. A simple main effects analysis showed statistically significant differences (p<0.05) in z score performance between groups appearing by Block 8. Representative individual performance graphs are shown in Fig 3. It was noted that behavior predictive of awareness varied between individuals, with (5) subjects demonstrating awareness before Block 20, labeled as Early Learners (see Fig 3a), and (4) subjects demonstrating awareness after Block 20, labeled as Late Learners (see Fig 3b). It was also noted that all subjects in the NOEXP group demonstrated an overall decrease in latency suggestive of implicit sequential learning (see Fig 3c).

#### EEG results

**Fig 5:**
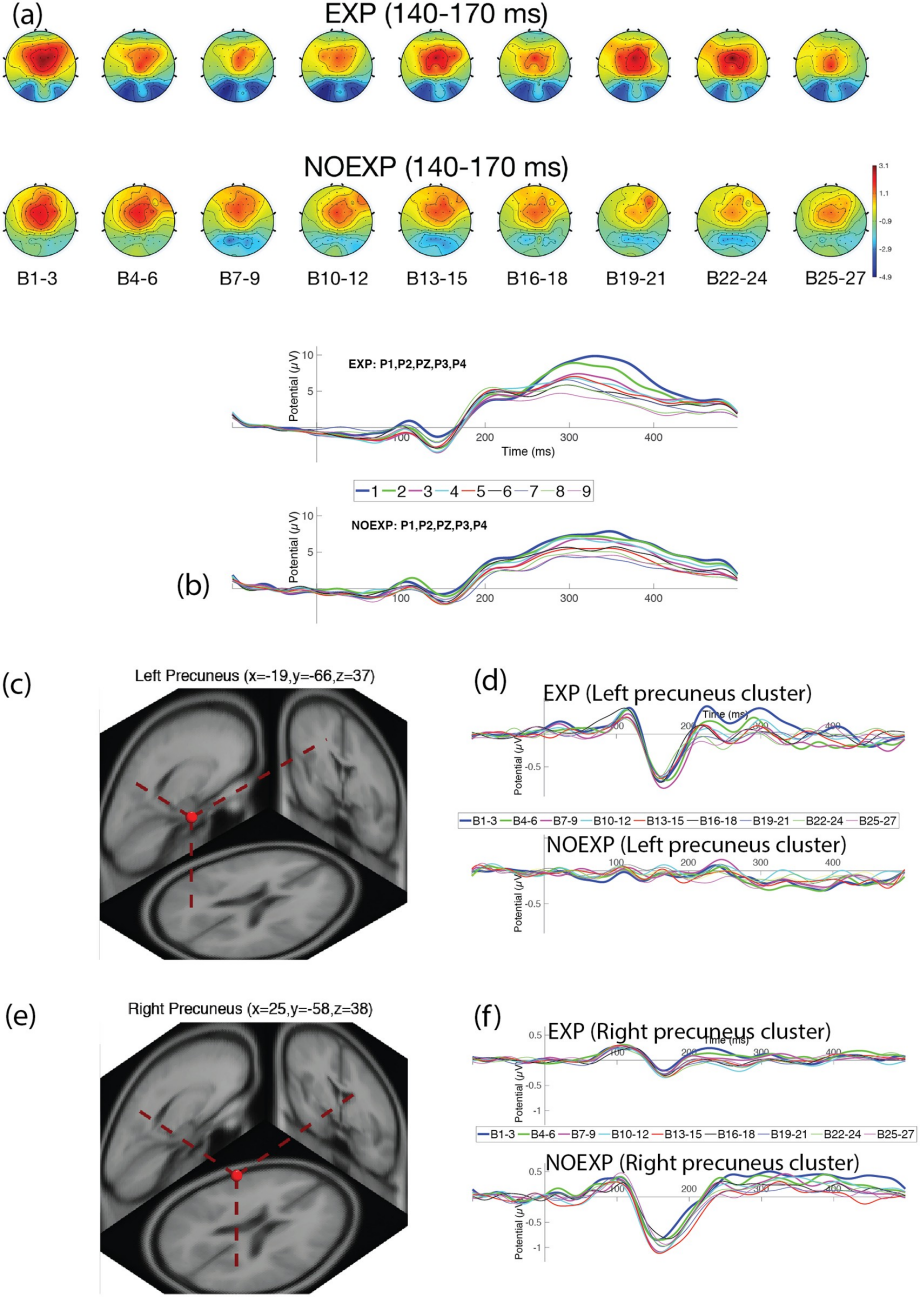
Neural activity for late N1 over parietal region. (a) Head maps for 9 periods of time during late N1 (140–170 msec). (b) ERP graph over parietal electrodes (P1, P2, P3, P4). (c) Dipole localization for late N1 component over left precuneus region. (d) ERP for left precuneus cluster. (e) Dipole localization for late N1 component over right precuneus region. (f) ERP for right precuneus cluster.

**Fig 6:**
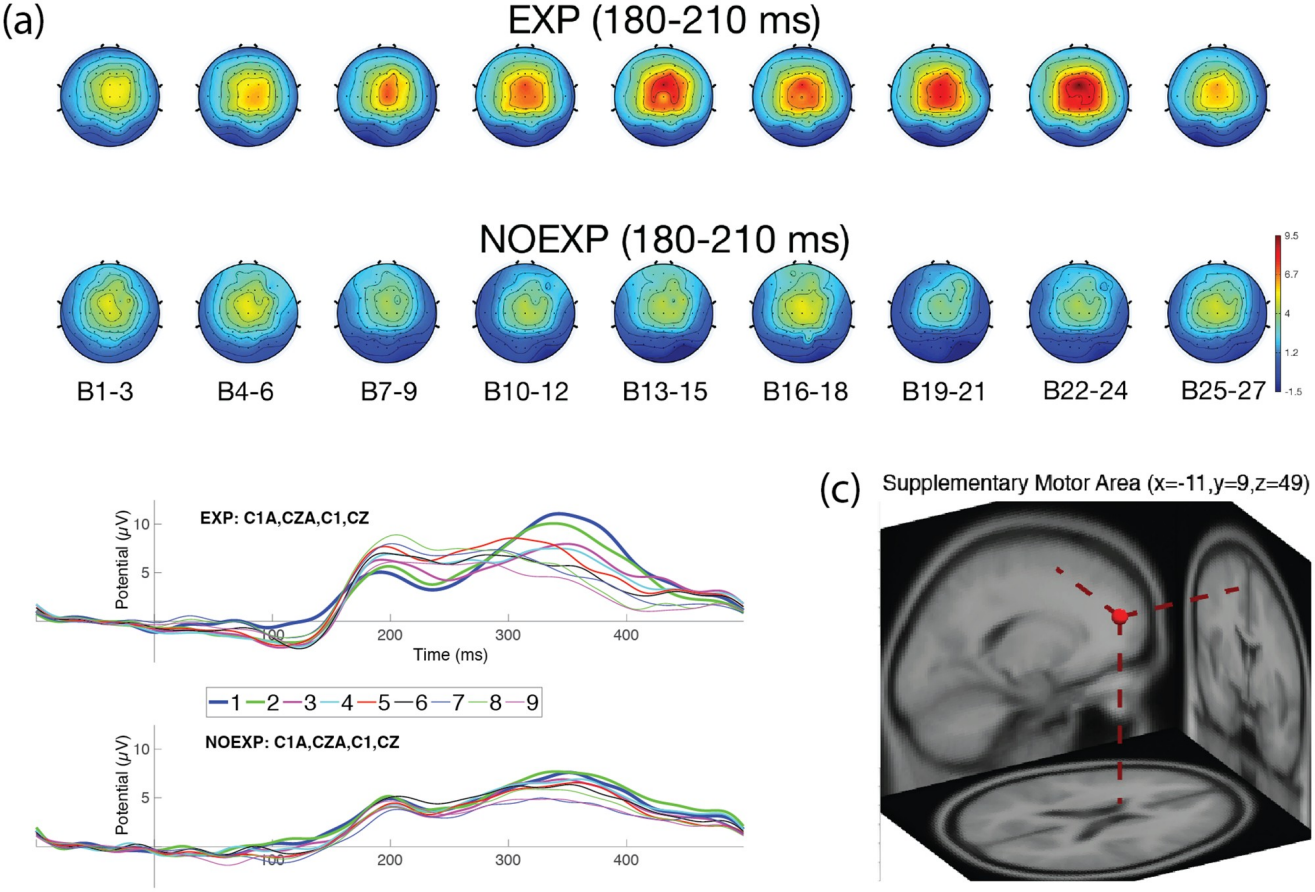
Neural activity for P2 over left frontocentral region. (a) Head maps for 9 periods of time during P2 (180–210 msec). (b) ERP graph over left frontocentral electrodes (C1A, CZA, C1, CZ). (c) Dipole localization for P2 component over left supplementary motor area.

**Fig 7:**
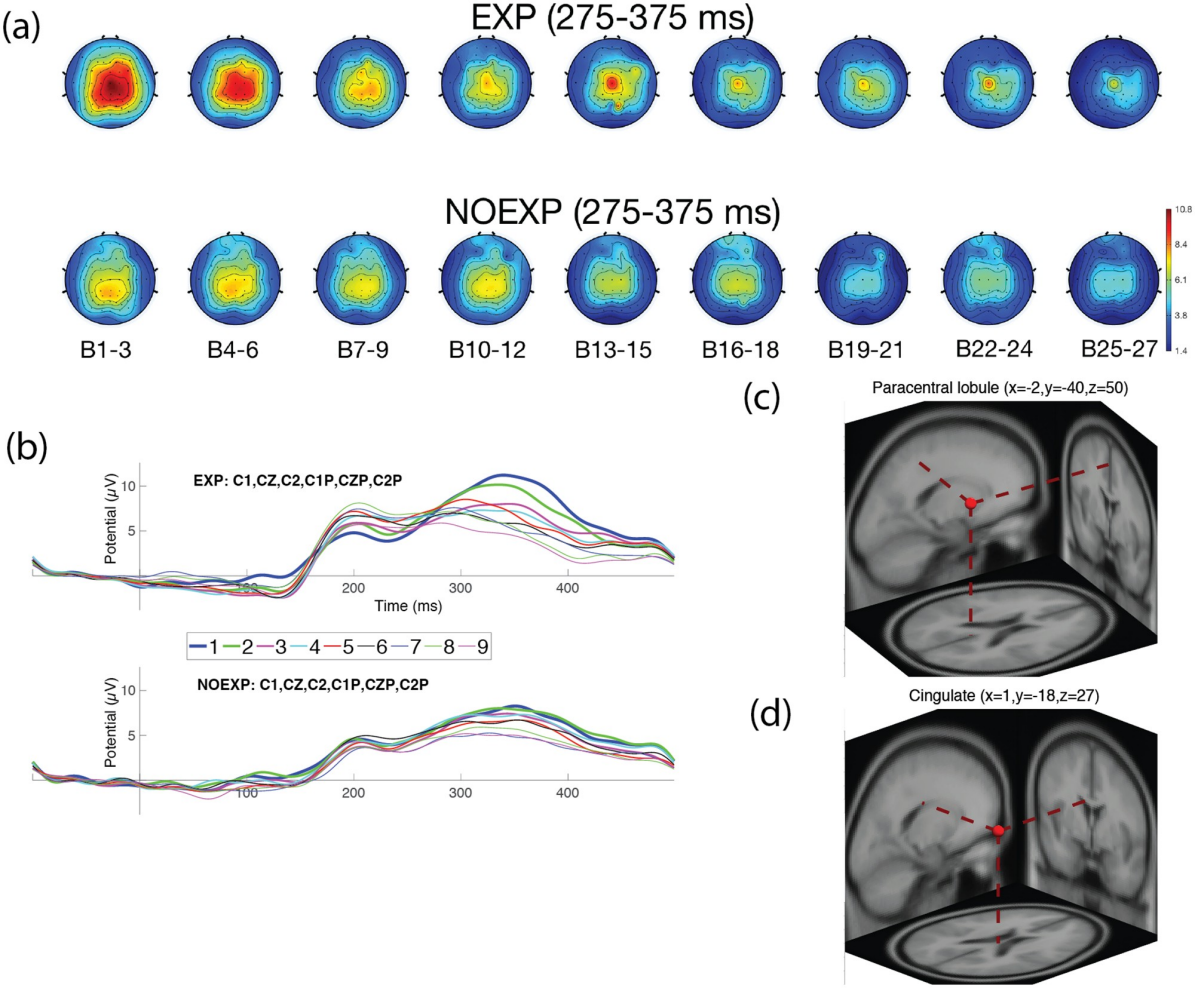
Neural activity for P3 over centroparietal region. (a) Head maps for 9 periods of time during P3 (275–375 msec). (b) ERP graph over centroparietal electrodes (C1, CZ, C2, C1P, CPZ, C2P). (c) Dipole localization for P3 component over paracentral region and (d) cingulate cortex.

EEG data for one subject was unable to be collected, so neural results were available for 20 subjects. Two of the subjects were falsely classified as NOEXP, and labeled as false negative (FN) and were excluded from further EEG statistical analyses, resulting in a final number of 18 subjects for EEG analysis, with (9) EXP and (9) NOEXP subjects. Figs 4–7 show head map topographies, ERP traces and dipole localizations for the ERP time periods of interest over the course of the experiment. For simplicity of visualization, images demonstrate activity over 9 sections of time, with 3 blocks combined in each time section.

#### Early N1 component (100–140 ms)

Fig 4a shows the head map activity changes over the experiment and Fig 4b shows the Early N1 over frontal electrodes (F1, FZ, F2) for both EXP and NOEXP groups. A repeated measures ANOVA for TIMExAWARENESS effect on early N1 peak amplitude demonstrated a main effect of time (F(8, 64) = 3.3795, p = 0.011) and awareness (F(1, 8) = 5.3712, p = 0.051), with no interaction effect (F(8, 64) = 1.3691, p = 0.2745). Post hoc analysis demonstrated the EXP group N1 amplitude was significantly greater (p<0.05) than the NOEXP group for Blocks 4–6, 7–9, and 10–12. The EXP group demonstrated a significant difference in amplitude, relative to baseline, by Blocks 4–6, while the NOEXP group did not demonstrate a significance difference from baseline until Blocks 13–15 (see Fig 4a). The EXP group demonstrated peak activations at Blocks 16–18, followed by a secondary peak at Blocks 25–27, showing similar clustering to the Early and Late Learner behaviors. Dipole localization results revealed a cluster localized to the anterior cingulate cortex (ACC) correlating with the early N1 component (see Fig 4c for dipole centroid image and Table 4 for Talairach coordinates and cluster distribution).

**Table 4:** Talairach coordinates and clustering values for source localization.

ERP Component	Dipole Localization	Talairach coordinates (x, y, z)	Residual variance	Percent of subjects included in cluster	EXP/NOEXP representation in cluster
*EarlyN*1	Anterior cingulate (BA 24)	(−4, 29, 7)	7.82%	38.9%	(4) EXP (3) NOEXP
*LateN*1	Right precuneus (BA 7)	(25, −58, 38)	5.12%	72.2%	(8) EXP (5) NOEXP
*LateN*1	Left precuneus (BA 7)	(−19, −66, 37)	4.58%	66.7%	(5) EXP (7) NOEXP
*P*2	Medial frontal gyrus (BA 6)	(−11, 9, 49)	7.18%	88.9%	(9) EXP (7) NOEXP
*P*3	Paracentral lobule (BA 5)	(0, −41, 52)	7.68%	44.4%	(2) EXP (6) NOEXP
*P*3	Precuneus (BA 7)	(−3, −46, 47)	7.52%	44.4%	(4) EXP (4) NOEXP
*P*3	Cingulate (BA 23)	(1, −18, 27)	8.00%	61.1%	(8) EXP (4) NOEXP

#### Late N1 component (140–170 ms)

Fig 5a shows the head maps demonstrating activity changes over time, while Fig 5b shows ERP changes over the parietal lobe electrodes (P1, P3, P2, P4). Repeated measures ANOVA for TIMExAWARENESS demonstrated no main effect of time (F(8, 64) = 0.5811, p = 0.817), or awareness (F(1, 8) = 1.0328, p = 0.374), with an interaction effect nearing significance (F(8, 64) = 2.1245, p = 0.077). Post hoc analysis showed the EXP group had a significant difference in amplitude, relative to baseline, by Blocks 4–6, while the NOEXP group demonstrated a significant difference by Blocks 7–9. The NOEXP group maintained a relatively stable N1 for the remainder of the experiment, while the EXP group demonstrated a reduced N1 amplitude toward the end of the experiment, reaching levels similar to baseline by Blocks 19–21. ICA cluster analysis identified two clusters correlating with the late N1 component, with dipole localizations at the left and right precuneus (see Fig 5c and 5e for dipole centroid images and Table 4 for Talairach coordinates and cluster distribution). Cluster ERP’s revealed a larger late N1 for the EXP group over the left precuneus, while the NOEXP group demonstrated a larger late N1 component over the right precuneus (see Fig 5d and 5f). Based on the ERP differences noted between the left and right parietal dipole clusters, an additional repeated measures ANOVA for TIMExAWARENESS was made for the right (P2 & P4) and left (P1 & P3) parietal electrodes separately. The right parietal electrodes showed no main effect of time (F(8, 64) = 0.6419, p = 0.739), or awareness (F(1, 8) = 0.1.1044, p = 0.347) and no interaction effect (F(8, 64) = 1.5351, p = 0.206). The left parietal electrodes also showed no main effect of time (F(8, 64) = 0.6639, p = 0.769) or awareness (F(1, 8) = 0.9479, p = 0.404). However, there was an interaction effect seen (F(8, 64) = 2.7751, p = 0.024), with EXP subjects demonstrating a significant difference in amplitude, relative, to baseline, by Blocks 4–6, while the NOEXP group did not show a significant change in amplitude.

#### P2 component (180–210 ms)

Fig 6a shows head map changes for the P2 time period demonstrating the peak activity at Blocks 13–15 and 22–24. The P2 ERP component (see Fig 6b) showed no significant main effect of time (F(8, 64) = 1.1908, p = 0.392) or awareness (F(1, 8) = 1.6912, p = 0.247), but did reveal a significant interaction effect (F(8, 64) = 3.7882, p = 0.002). Post hoc analysis revealed the EXP group had a significant difference in peak amplitude, relative to baseline compared to the NOEXP group. The largest significant differences were noted to occur at Blocks 13–15 and Blocks 22–24, again clustering in two time periods similar to the Early and Late Learner behaviors. The NOEXP group did not demonstrate a significant change from baseline over the course of the experiment. The ICA cluster correlating with the P2 component showed a dipole localization at the left supplementary motor area (SMA) as shown in Fig 6c (refer also to Table 4 for Talairach coordinates and cluster distribution).

#### P3 component (275–375 ms)

Head maps in Fig 7a show that both groups demonstrated a steady decline in peak amplitude over the course of the experiment. Peak amplitude for the P3 ERP component over centroparietal electrodes (see Fig 7b) revealed a significant main effect of time (F(8, 64) = 8.6358, p = 0.0035), but not for awareness (F(1, 8) = 0.2052, p = 0.698), with no interaction effect (F(8, 64) = 1.1135, p = 0.405). Post hoc, pairwise comparison, revealed a significant difference (p<0.05), from baseline, by Blocks 13–15 for the EXP group and by Blocks 19–21 for the NOEXP group. Two clusters correlating with the P300 component showed dipole localization at the paracentral region (see Fig 7c) and the cingulate cortex (see Fig 7d). Separation of dipole localizations, by awareness, indicated localization for EXP subjects at precuneus/ cingulate cortices, and paracentral/cingulate localizations for NOEXP subjects. See Table 4 for Talairach coordinates and cluster distribution).

#### Neurobehavioral correlation analysis between timing of EXP behavior and peak ERP component amplitude

Correlational analysis between timing of individual behavior (drop below threshold for EXP and lowest z-score for NOEXP) and peak ERP component amplitude activity was examined over the course of all 27 blocks. Neurobehavioral correlational results for each ERP component, with Spearman correlation rho values, are summarized in Table 5. For the early N1, there was a significant correlation for the EXP group (rho = 0.68, p = 0.041), but not for the NOEXP group (rho = −0.16, p = 0.684). There was no significant correlation for either group with the late N1 component over right parietal (EXP: rho = −0.09, p = 0.811 and NOEXP: rho = 0.29, p = 0.448). The left parietal correlation results revealed a significant correlation for EXP subjects (rho = 0.82, p = 0.007), but not for NOEXP subjects (rho = −0.33, p = 0.378). The P2 also demonstrated a strong correlation for the EXP group (rho = 0.82, p = 0.006), with no significant correlation for the NOEXP group (rho = −0.39, p = 0.298). The P3 component over centroparietal electrodes, and posterior cingulate dipole localization, did not reveal significant correlations for either group, although EXP subject correlation was nearing significance (EXP: rho = 0.62, p = 0.076 and NOEXP: rho = −0.14, p = 0.726).

**Table 5:** Spearman significance values for neurobehavioral correlations.

	Early N1:ACC		Late N1: Left Parietal		Late N1: Right Parietal		P2:SMA		P3:PCC	
*Group*	EXP*	NOEXP	EXP**	NOEXP	EXP	NOEXP	EXP**	NOEXP	EXP^†^	NOEXP
*Rho*	0.69	-0.16	0.82	-0.33	-0.09	0.29	0.82	-0.39	0.62	-0.14
*pvalue*	0.041	0.684	0.007	0.378	0.811	0.448	0.006	0.298	0.076	0.726

^†^p<0.1;

*p<0.05;

**p<0.01

#### Neurobehavioral correlation analysis: Change in latency over time relative to change in P3 amplitude over time

**Fig 8:**
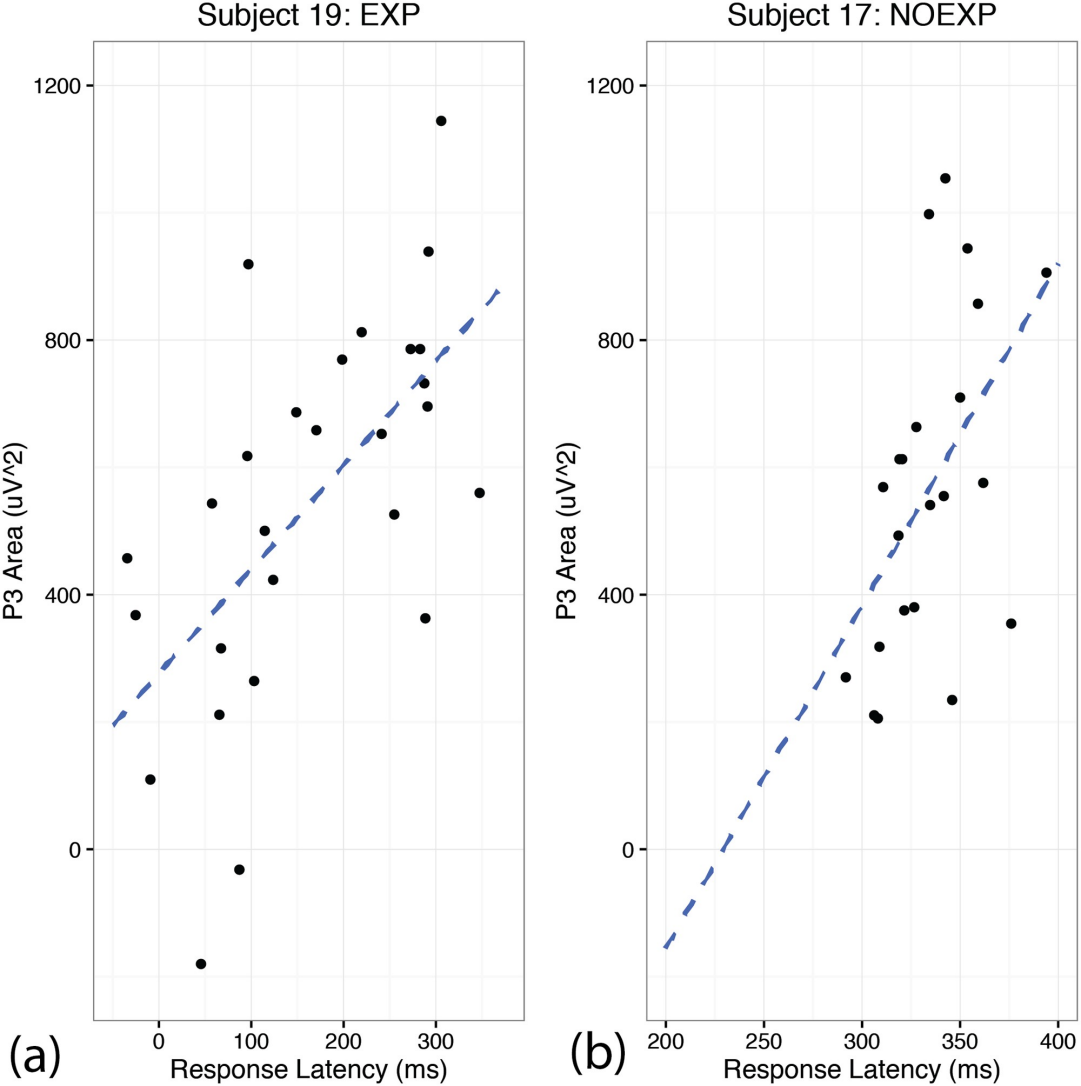
Representative individual correlation plots of P3 amplitude with latency. Correlation of P300 amplitude with response latency for each block. Note the strong correlation regardless of awareness level.

Due to the localization of the P3 over sensory association and cingulate regions, along with the common overall decrease in amplitude noted for both EXP and NOEXP groups, it was hypothesized that the P3 may represent adaptation to the paradigm not specific to the presence awareness. As both the P3 amplitude and sequence response latency decreased for all subjects over the course of the experiment, an additional correlational analysis was conducted between the P3 amplitude and subject mean response latency for each block to further explore the neurobehavioral relevance of the significant change in P3 amplitude seen in both groups. A mixed model analysis with fixed effects for latency and awareness, and random effects of subject intercept and slope revealed a significant relationship of P3 amplitude and latency (t(442) = 2.719, p = 0.0068) and a significant main effect of awareness (t(16) = 2.660, p = 0.0171) with no interaction effect. EXP subjects had a greater mean intercept (335.47 ± 94.31 uV^2^) compared to NOEXP subjects (84.64 ± 136.15 uV^2^). Fig 8 shows two representative individual correlation plots, one from each group.

#### Neural correlation analysis between timing of peak ERP component amplitude

**Fig 9:**
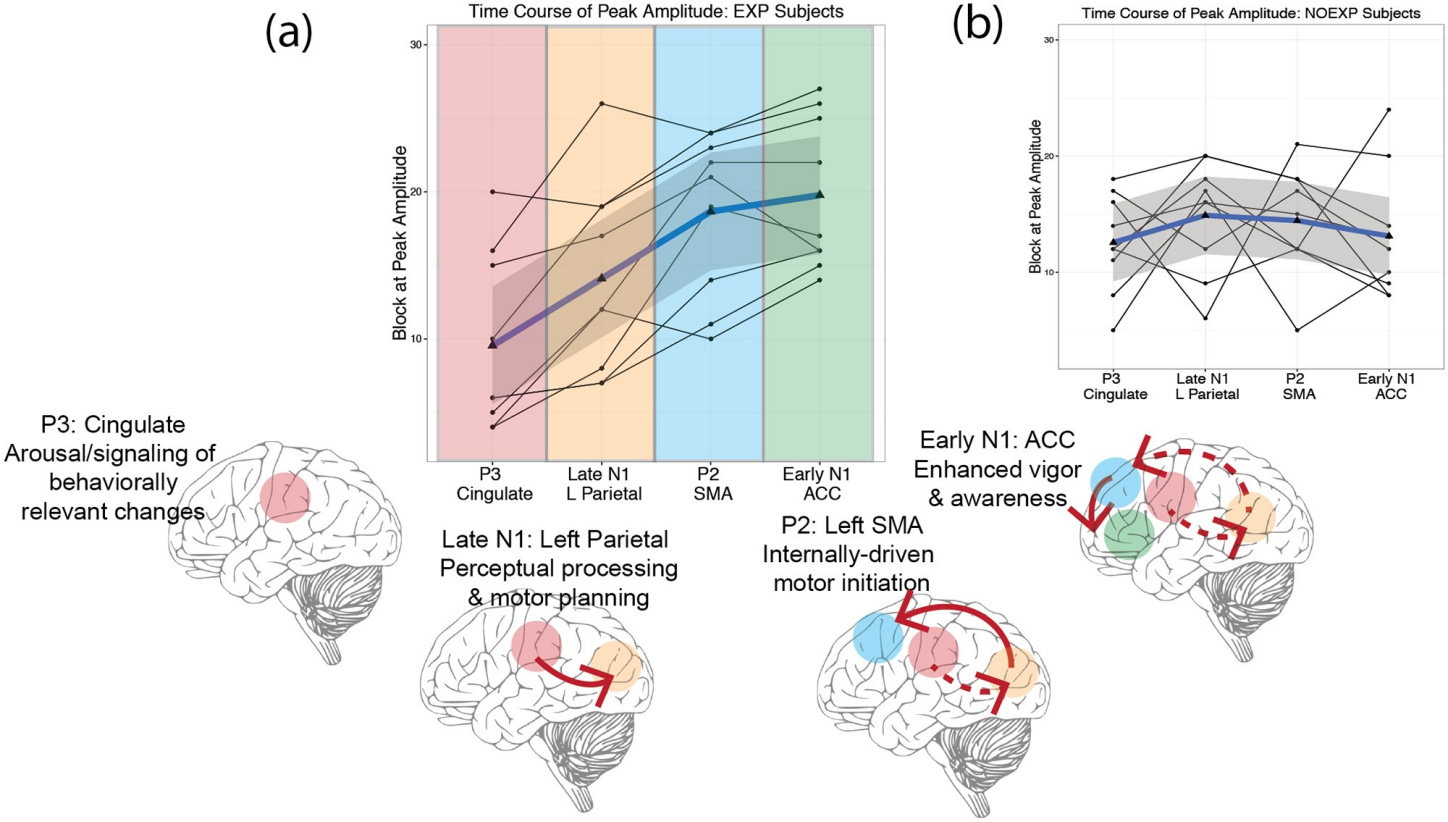
Timing of peak ERP component occurrences. (a) EXP timing with proposed timing of peak activations required for development of incidental explicit awareness. (b) NOEXP timing. Note the seemingly random progression of peak activation between subjects.

As all ERP components demonstrated significant, or near significant, correlations with EXP behavior, an additional correlational analysis was conducted for the timing of each ERP component. Neural correlations between ERP components in regions demonstrating significant correlation with behavior demonstrated significant correlations between the early N1, left late N1, P2 and posterior cingulate P3 for EXP subjects that was not observed for NOEXP subjects. Spearman correlation values are summarized in Table 6. Timing of the peak occurrences for these 4 regions in EXP subjects is plotted in Fig 9. A mixed model analysis for timing of peak amplitude occurrence with fixed effect of ERP component order (P3, Late N1, P2 and Early N1) and random effect of subject revealed a significant difference in the timing of the P3 peak amplitude (t(48) = 6.98, p<0.001) with EXP subjects demonstrating the peak P3 component 3 blocks earlier (EXP: Block 9.55 ± 6.00, NOEXP: Block 12.55 ± 4.24). There was no significant relationship between timing of Late N2, P2 or Early N1 components and P3 peak timing (LateN1: t(48) = 1.24, p = 0.221, P2: t(48) = 1.00, p = 0.321, EarlyN1: t(48) = 0.295, p = 0.769). Adding the fixed effect of awareness did not significantly improve the model (*X^2^*(7, 1) = 0.911, p = 0.339), but the addition of an interaction effect resulted in significant model improvement (*X^2^*(10, 3) = 16.14, p = 0.002). Due to the interaction effect, a separate repeated measures analysis was conducted for EXP and NOEXP subjects, utilizing Tukey contrasts for multiple comparison of means. The NOEXP subjects demonstrated no significant linear trends between ERP component peak timing. The EXP group, however, demonstrated a linear trend between P3, LateN1 and P2, leveling off at EarlyN1 as shown in Table 6 and Fig 9.

**Table 6:** Correlation matrix for EXP and NOEXP groups shown in Fig 9.

Linear Hypotheses	EXP	NOEXP
*LateN*1 − *P*3 = 0	p = 0.007	p = 0.747
*P*2 − *P*3 = 0	p < 0.001	p = 0.140
*EarlyN*1 − *P*3 = 0	p < 0.001	p = 0.994
*P*2 − *LateN*1 = 0	p = 0.007	p = 0.998
*EarlyN*1 − *LateN*1 = 0	p < 0.001	p = 0.913
*EarlyN*1 − *P*2 = 0	p = 0.860	p = 0.914

## Discussion

The current study sought to identify and validate a reliable behavioral predictor for the presence of incidental explicit awareness in a sequential motor task. This type of incidental discovery may be reflective of the neuronal interactions occurring during the exploratory motor learning often utilized in rehabilitation. Recent studies examining such incidentally developed explicit awareness show benefits of enhanced perceptual sensitivity and increased motivational vigor [7, 15], but have not attempted to identify when such awareness occurred. A major challenge for such indicators is detecting the presence of awareness without interfering with the incidental nature of the discovery. Results from Experiment 1, provided an individualized indicator, with neurobehavioral validation in Experiment 2. Our work showed frontoparietal activations in EXP subjects, demonstrating a facilitative, learning-dependent interaction between perceptual and motor regions specific to explicit sequential motor learning.

### Why the model works

The proposed behavioral indicator utilizes the dramatic decrease in latency observed for subjects expressing explicit awareness [16, 17]. Previous studies examining incidental awareness predictors utilized tasks focused on single event occurrences, but it is unclear how well their method translates to sequential motor tasks where multiple levels of insight are needed for full discovery [16]. Our indicator addresses this concern by examining behavior over each sequence repetition, identifying when a subject is aware of the entire sequence. Utilizing baseline performance accounts for initial variability in visuomotor performance, allowing for an individualized approach in awareness identification [9]. In the initial development of the threshold indicator for Experiment 1, a baseline of the first 45-key presses were used. However, for the 7-element condition, this included 6 sequence repetitions which may have resulted in some subjects developing some level of awareness by the end of the baseline period. This potential confound was addressed in Experiment 2 using a random baseline for threshold calculation. The resulting identified threshold indicator of two consecutive sequence performances below a relative z-score of −1.85, showed 81.8% sensitivity and 100% specificity for subjects in Experiment 2.

### Temporal course of neural activations with explicit awareness

Peak amplitude analysis of ERP components demonstrates a consistent temporal course of neural activity, suggesting involvement of parieto-frontal areas in perceptual processing and subsequent motor execution of the visuomotor task. Results showed significant correlations between timing of predicted EXP awareness and peak amplitude timing over cingulate, left parietal, premotor and prefrontal regions. The sequential peak activation of these areas suggest a facilitative neural network associated with development of incidental explicit awareness in sequential motor learning. Such learning-dependent changes have been noted with visual and somatosensory perceptual learning [32, 33], but have not examined the impact of such learning on motor tasks. Studies utilizing motor tasks typically assess learning-dependent changes over multiple sessions [34–36]. This is the first study, to our knowledge, to examine both perceptual and motor related learning-dependent changes within a single session.

EXP subjects showed a strong linear trend for the timing of peak activations in a frontoparietal network, progressing from posterior cingulate to left precuneus, left supplementary motor area (SMA) and anterior cingulate (ACC). The presence of peak activations in ERP components may be indicative of optimized neuronal communication through mechanisms such as enhanced synaptic efficiency reflective of Hebbian learning [32] or altered communication patterns increasing task-relevant neuronal activity and reducing activity in task-irrelevant neurons [37]. This learning may involve similar mechanisms as proposed for the development of predictive mirror neurons which utilize spike-timing-dependent plasticity and are suggested to require both consistently causal and contingent neuronal interactions [38]. Causality requires consistent presynaptic firing within a time window on the order of tens of milliseconds [39], potentially reflected in the progression of the amplitude of individual ERP components. For learning to occur, however, causality must also be accompanied by contingency, the determination of whether the presynaptic activity is informative about postsynaptic activity, and functions under a time window of minutes [40]. The ERP components associated with each region indicates differential timing and separate roles in the processing and decision-making aspects of integrating the stimulus-response mapping, providing both a causal and contingent interaction between perceptual processing and motor actions. As proposed below, this consistent causality and contingency may provide the mechanism allowing a person to shift from a reliance on external stimuli for movement execution to an internally-generated learned motor sequence.

The first peak activation for EXP subjects, occurred with the P3 which localized to the cingulate and precuneus. The P3 is attributed with engagement of attention and novelty processing [29], while the cingulate is involved in attentional arousal and signaling of behaviorally relevant changes in stimuli [41], and the precuneus with perceptual decision-making [42]. The accumulation of visual and somatosensory evidence over time may result in enhanced synaptic communication within cingulate neuronal pools, indicative of the neural recognition of a behaviorally relevant regularity in the environment. Reaching peak activation may alter attention over bilateral precuneus regions to include an external focus on visual stimuli, and an internal focus to assist in integrating visuospatial information with motor execution. It is unclear, from the current data, what variables contribute to EXP subjects utilizing peak P3 activity in a facilitative manner for sequence learning, while NOEXP subjects failed to do so.

The inclusion of an internal focus is reflected in the subsequent peak activation of the late N1, over the left precuneus region. This component is attributed to perceptual processing of stimuli [43], often localizing over posterior parietal cortex, including the precuneus. Functionally, the precuneus has been shown to demonstrate a hemispheric specialization with visuomotor tasks. The right precuneus is attributed to stimulus encoding and visuospatial transformations necessary for eye-hand coordinated movements [42, 44, 45], while left precuneus activation appears to be involved with praxis planning and encoding of future intentions [46–48]. The enhanced left precuneus activation observed for EXP subjects provides evidence for an attentional resource shift to include the left parietal region. This addition in attentional resources may allow for sensorimotor integration of visual stimuli information with finger movement dynamics leading to development of an internal representation of the visuomotor relationship [49]. This visuomotor relationship may be used as a predictive motor plan which can assist in accumulating probabilistic data to test the possible regularity in visual stimuli or motor responses signaled by the PCC. Future studies could seek to identify if probabalistic search is a feature of this phase in learning. Magnitude changes in the error-related negativity (ERN) ERP component has been shown to be enhanced when expectations are violated [23] and could be a useful neural marker of when a subject is testing predictions of stimuli locations. Utilization of eye-tracking to identify anticipatory eye movements, both correct and incorrect, could provide a method to assess if false predictions of stimulation locations (through an ERN response) may be correlative with the timing of the late N1 peak. Alternatively, one could utilize the onset of a peak late N1 as an individual marker for when to begin imposing “oddball” stimuli to the sequence presentation and assess the magnitude of ERN response relative to random stimuli. Unlike the left precuneus, the right precuneus did not show a significant correlation with behavior, suggesting that subjects continued to perform visuospatial transformations regardless of awareness. Although, as explained below, the function of such visuomotor transformations may shift as awareness develops.

After left parietal areas demonstrate peak activity, there is a concurrent peak activation over left SMA and ACC regions, in the P2 and early N1 components, respectively. The P2 is known to be sensitive to tasks comparing sensory inputs and presence of conscious awareness [27, 50–52], while the SMA has long been associated with internally-guided movements [25]. The progressive increase in SMA activity, therefore, may reflect enhanced attentional resources for initiation of movement patterns less dependent on the external stimulus. While both EXP and NOEXP subjects demonstrated dipole activation over the SMA, EXP subjects showed a much greater increase in amplitude, with peak occurrence being highly correlated with explicit behavior. This correlation provides evidence for a shift in EXP subjects to the use of internally generated movements, accompanied by a conscious awareness of these movements. The current study utilized reaction time as the behavioral measure, but recent studies by Moisello et al. have demonstrated that awareness is accompanied not only by changes in overall reaction time, but also changes in movement time and movement patterns [10, 53]. Both studies present data that suggests the value of anticipatory movement onset with awareness may be to provide additional time for more precise movements. To further elucidate the motoric significance of the P2 peak, it would be valuable to examine correlations of the P2 peak with additional measures such as movement onset, movement time, touch duration or movement peak velocity.

Peak activation over ACC regions during the early N1 timeframe may reflect a neural representation of enhanced attentional resources involved in explicitly executing the sequence [4] and the enhanced motivation observed with explicit awareness [15]. The early N1 component is typically associated with attentional gating [54, 55], while the ACC is attributed to multiple cognitive roles such as decision-making, reward, learning and consciousness [56]. The occurrence of peak activity for the ACC and SMA regions suggest reciprocal communication between anticipatory visuospatial confirmation of the visual cue and facilitation of predicted motor behavior as conscious awareness of the sequence develops. Examination of gaze patterns during awareness development may help to further elucidate the role of anticipatory eye movements [55] in providing confirmatory feedback.

### Neural activations without explicit awareness

NOEXP subjects did not show any significant correlations in peak activity. This group demonstrated an irregular sequence of regional peak activation between subjects, indicating a decoupling in peak timing between the cingulate arousal, perceptual integration of stimulus-response and internalization of motor execution. The lower amplitude of left precuneus late N1 activity, suggest that NOEXP subjects fail to make the attentional shift, resulting in a retained reliance on the external visual cues for motor initiation. Without the transition to enhanced left parietal activity, NOEXP subjects may have been unable to form an internal representation of the sequence pattern, preventing the shift to internal movement execution and development of conscious awareness of movement patterns.

### Limitations

The current study indicates that adaptive neuronal changes in perceptual processing occur in subjects developing awareness incidentally. It is unclear, however, whether these learning-dependent changes are not occurring in NOEXP subjects, or if they did not have enough time for facilitative neuronal interactions needed for awareness to occur. Another limitation is the presence of two false negative classifications. This may be related to the use of reaction time as the outcome measure. Other behavioral measures, such as kinematics, may provide additional information beneficial in accurately classifying these subjects as explicitly aware. Having a behavioral measure which identifies these subjects would provide further opportunities to explore any variations in neural activation patterns utilized during the, seemingly, more controlled motor execution for these subjects.

## Conclusions

The results of the current study provide an indirect, individualized, behavioral indicator for the detecting development of explicit awareness in a sequential motor task. The individual nature of the indicator provides a tool for monitoring progress in rehabilitative, exploratory motor learning paradigms. Utilization of the proposed indicator with EEG recordings provides neurobehavioral evidence of a learning-dependent, facilitative fronto-parietal network involved with the development of explicit awareness in a sequential motor task. This incidentally-driven facilitative network may provide valuable lines of research for rehabilitative programs using exploratory learning. Additionally, the apparent pivotal role of enhanced cingulate activity may help explain sequential learning detriments seen with amputees, stroke patients and with aging [1–3]. In sum, our results indicate learning-dependent changes in a fronto-parietal network facilitate changes in perceptual processing to enhance motor learning.
